# Association of socio-economic status with dietary habits and anemia prevalence in the geriatric population

**DOI:** 10.6026/973206300220759

**Published:** 2026-02-28

**Authors:** Sharanya Kandaswamy, Cibi Kannan, Jeevithan Shanmugam, Karthikeyan T.M, Balaji Venkatesan

**Affiliations:** 1 Department of Pathology, KMCH Institute of Health Sciences and Research, Coimbatore, India; 2 Department of General Medicine, KMCH Institute of Health Sciences and Research, Coimbatore, India; 3 Department of Community Medicine, KMCH Institute of Health Sciences and Research, Coimbatore, India; 4 KMCH Institute of Health Sciences and Research, Coimbatore, India

**Keywords:** Anemia, geriatric population, socio-economic status, dietary habits, dietary diversity, nutrition, Modified Kuppuswamy Scale

## Abstract

Socioeconomic status (SES) strongly influences food choices, dietary diversity and healthcare access, potentially increasing anemia prevalence in geriatric individuals. Therefore, it is of interest to examine the association between socioeconomic status (SES), nutritional habits and anemia prevalence among 336 geriatric individuals aged ≥60 years. SES was assessed using the Modified Kuppuswamy Scale (2024), alongside dietary habits and hemoglobin levels. Anemia prevalence was 48.2%, with low SES showing significantly higher rates (61.4%) than middle (44.8%) or high SES (29.6%) groups (p < 0.001). Dietary inadequacies were markedly more common in the low SES group. This study advances knowledge by providing India-specific evidence linking graded SES differences to anemia and poor nutrition in older adults, guiding targeted interventions.

## Background:

Worldwide, there is a significant shift in the demographic trends. As the population ages, the number of people aged 60 and older will increase significantly. By 2030, it is projected that there will be 1.4 billion people aged 60 or older [1]. These aging populations will predominantly affect low and middle-income countries. Approximately 80% of the world's older adults will reside in developing (low- and middle-income) countries by 2050 [2]. Anemia is still a significant public health concern; at this time, it is estimated that almost 1.8 billion people throughout the world, across all ages, have anemia [3]. Although research and public health initiatives have focused primarily on children and pregnant women, older adults are an increasingly but largely unrecognized group who are at risk for developing anemia [4]. The elderly may be at higher risk of anemia due to multiple factors, including decreased nutrient intake, a range of chronic conditions they may be suffering from, chronic inflammation and reduced nutrient absorption and utilisation, which develop as people age [5]. Another major factor influencing the likelihood of becoming anaemic is the associated Economic Status (SES) of individuals and its effect on the availability of nutritious food items in the economy which can limit their intake of essential micronutrients like iron, vitamin B12 and folate (micronutrients needed for appropriate red blood cell production) as well as limit an individual's ability to respond to treatment when diagnosed with anaemia [6]. There are considerable variations in dietary patterns across SES segments within many developing countries, including India.

Many people consume primarily plant- or cereal-based foods, which result in inadequate levels of essential bioavailable micronutrients (such as iron, vitamin B12 and folate) needed for adequate red blood cell production [7]. There is limited information available on how Economic Status (SES) interacts with dietary habits and risk factors for anemia among older adults living in India; on the other hand, considerable amounts of anemia data have been collected at the National Level. Therefore, it is of interest to investigate the association between socio-economic status, dietary patterns and the prevalence of anemia among older adults (age ≥ 60 years) who visited a tertiary care facility in India.

## Methods:

## Study design and setting:

A cross-sectional observational study was conducted from July 2024 to June 2025 at the KMCH Institute of Health Sciences and Research, a tertiary-care training hospital serving urban and semi-urban communities in South India. This study focused on both outpatient and inpatient departments to obtain a comprehensive representation of geriatric patients.

## Study population:

The geriatric population comprised individuals aged 60 years or older who utilized medical services during the study period. Participants were recruited for the study via convenience sampling due to logistical constraints and the various sources of clinical inflow for geriatric patients.

## Sample size determination:

The sample size for the present study was calculated at 336 participants. Sample size calculations were based on the findings of Retnakumar *et al.* (2020), who reported a prevalence of anemia among geriatric individuals of 68.3% [8]. Therefore, the odds ratio of 2.842 indicates that geriatric individuals have a greater risk of developing anemia due to low consumption of dark green leafy vegetables. Using a regression-based methodology for sample size estimation will determine that a minimum of 112 subjects is necessary. Once the design effect of 3 was added to obtain the representative sample, the final sample size is 336.

## Inclusion criteria:

[1] Age ≥60 Years

[2] Willing to Give Written Informed Consent

[3] Have been diagnosed with anemia according to the World Health Organization (WHO) definition (Haemoglobin levels < 13 grams/dL for men and < 12 grams/dL for women).

## Exclusion criteria:

The following conditions will lead to exclusion from this study to minimize confounding factors:

[1] Chronic Kidney Disease (CKD)

[2] Received a blood transfusion within the previous 3 months

[3] Gastrointestinal Disorders That Affect Nutrient Absorption,

[4] Currently taking Iron Supplementation or Erythropoietin Therapy,

[5] Dental Plate Complete that May Affect the accuracy of dietary Information Provided

[6] Cognitive Impairments that impair the ability to participate in the research.

[7] Participants who are not willing to participate or cooperate

## Data collection procedures:

[1] Haemoglobin estimation: To estimate haemoglobin levels, an automated haematology analyser was used to determine whether subjects were anaemic according to the WHO classification.

[2] Socio-economic Status (SES): SES was evaluated using the Modified Kuppuswamy Scale (2024) [9], which considers an individual's educational attainment, occupation and family income.

[3] Dietary assessment: Individual nutritional patterns were assessed by asking subjects to complete a 24-Hour Dietary Recall, a Food-Frequency Questionnaire and an assessment of the timing of meals, how diverse a person's diet is and how many iron-containing foods they consume.

## Statistical analysis:

Standard statistical software was used to analyse the data collected from the previous steps. Baseline characteristics were presented using descriptive statistics (i.e., percentages, means, standard deviations) and the association between SES, dietary patterns and anemia was examined using the Chi-Square test. The significant predictors of anemia were determined using binary logistic regression, with p-values < 0.05 considered statistically significant.

## Results:

Table 1 provides a summary of the demographic information for the 336 geriatric patients who participated in the study, including the distribution of patient ages by gender and their residence category. Table 2 provides an overview of anemia prevalence among geriatric patients. 48.2% of this population has anemia, with an average haemoglobin concentration. Table 3 compares the socio-economic status of patients with low socio-economic status and its relationship with anemia prevalence. An elevated level of anemia prevalence is seen among individuals with low SES. In Table 4, we compare dietary consumption across three SES levels. It is pretty clear that people with lower SES typically consume diets with significantly lower dietary diversity and lower intake of iron-rich foods. Table 5 illustrates a clear link between low dietary diversity and a higher prevalence of anemia. Respondents diagnosed with anemia consumed foods rich in micronutrients at substantially lower levels than did non-anaemic individuals. Table 6 reveals that, through logistic regression modelling, being classified as low SES and consuming a low-diversity diet each independently predicts the likelihood of anemia in older adults. According to Table 7, individuals classified as lower SES had statistically significantly lower haemoglobin levels than those classified as middle- or high-SES.

## Discussion:

An investigation was conducted to compare socio-economic status, dietary practices and iron deficiency among 336 older people to determine whether these factors were related. The results show a very high frequency of anaemia among the older generation (48.2%), which is consistent with previous studies that found that the prevalence of anaemia among the aged varies from 20% to 60%, depending on the location of the population, their eating habits and their incidence of chronic disease Katsumi *et al.* 2021 [10]. The average age of the participants in this study was 68.4 years, which previous studies have proved to coincide with the increase of anaemic cases among older populations as a result of decreased bone marrow function and the body's inability to absorb many of the nutrients that are needed [11]. The prevalence of anemia was strongly correlated with a person's income level (SES). The anemia rate among persons with low SES was nearly double that of their counterparts living in middle/high SES environments, at 61.4% compared to 44.8% for the middle SES group and 29.6% for the high SES group (p < 0.001). Studies that have identified this effect came from many different sources. A 2023 Indian study by Zhang *et al.* concluded that lower incomes are associated with lower micronutrient intake and a higher risk of anaemia in older adults [12]. Similarly, Lopes *et al.* (2022) determined that the availability of food at a price that is affordable based on your financial means for conducting your daily living expenses is directly correlated to a person's income level/S.E.S. Further evidence of the relationship between Low SES and Nutritional Anaemia has been generated from community- dwelling older adults [13]. Our findings were similar to those in this study: participants with low SES had significantly lower haemoglobin levels (11.2 g/dL) than their high-SES counterparts (12.6 g/dL). Additionally, the survey by Moradi *et al.* (2024) [14] found that the low-SES group relied more on financial assistance from others and had less access to good living conditions and food, both of which are commonly known to contribute to increased anemia. There was a significant difference in dietary diversity between low to low-socioeconomic status (SES) individuals versus individuals in higher SES categories. The average DDS for low SES individuals was 4.1 ± 1.0 compared to an average DDS of 6.3 ± 1.2 for higher SES individuals. Low-SES individuals had lower intake of GLVs, pulses, fruits and animal proteins, which supports previous research indicating that economic barriers can limit access to nutrient-dense foods [15]. The current research indicated that individuals with anemia also had a significantly lower DDS than their non-anaemic counterparts and showed a strong correlation between anemia and diet quality. A supporting study by Retnakumar *et al.* (2020) found that low GLV consumption was associated with a threefold increase in the risk of anemia among older adults [8]. Also, Greenblum *et al.* (2022) described a high prevalence of anemia among older populations in Mexico, China and South Africa (24-91%). They attributed some of the differences to differences in dietary patterns and socio-economic status (SES) [4]. In India, where the vast majority of the population consumes a primarily vegetarian diet (which contains little or no animal protein), this is particularly important because such a diet provides little or no iron. On average, older individuals need more nutrients than younger individuals and have a lower ability to absorb nutrients from food, which puts them at greater risk of developing an iron deficiency [16].

The use of logistic regression found that being in a low Socio-economic Status was found to be a strong independent risk factor for developing anemia (adjusted Odds Ratios = AOR 2.71; 95%Confidence Interval = CI 1.65 to 4.45) as well as having a Poor Dietary Diversity AOR (2.39; 95%CI 1.52 to 3.76) score was also found to be a strong, independent predictor of Anemia status. Notably, both conditions demonstrate that poor nutrition can be affected by socio-economic disadvantage. Likewise, Katsumi *et al.* (2021) [10] found that poverty and nutritional deficiency both account for a large share of anemia prevalence among the older adult population. The relationship between a reduced Dietary Diversity Score and the incidence of micronutrient deficiencies in seniors (including increased Iron, Folate and B12 deficiencies) has been established in multiple studies. Most recently, the systematic review conducted by Liu *et al.* 2024, found that a low Dietary Diversity Score (DDS) among older adults significantly increased the risk of micronutrient deficiencies in Iron, Folate and B12 [17].

## Conclusion:

Lower-SES individuals also exhibited less varied diets and higher deficiency rates of essential micronutrients: Iron, Folate and Vitamin B12. Inadequate levels of these micronutrients are thought to be crucial mediators connecting low SES with anaemia. Early identification of older adults at risk of dietary insufficiency, along with SES-related risk factors, combined with appropriate nutritional interventions, may help reduce the incidence of anaemia.

## Figures and Tables

**Table 1:** Baseline demographic characteristics of the study population (n = 336)

**Variable**	**Category/mean ± SD**	**Frequency (n)**	**Percentage (%)**
Age (years)	68.4 ± 6.1	-	-
Gender	Male	162	48
	Female	174	52
Residence	Urban	128	38.1
	Rural	208	61.9

**Table 2:** Prevalence of anemia among the study population

**Variable**	**Category**	**Frequency (n)**	**Percentage (%)**
Anemia status	Present	162	48.2
	Absent	174	51.8
Mean haemoglobin (g/dl)	Total	11.8 ± 1.4	-

**Table 3:** Socioeconomic status (SES) distribution and anemia prevalence

**SES category (modified Kuppuswamy)**	**Total (n)**	**Anemia present n (%)**	**Anemia absent n (%)**
Low SES	140	86 (61.4%)	54 (38.6%)
Middle SES	116	52 (44.8%)	64 (55.2%)
High SES	80	24 (29.6%)	56 (70.4%)
P-value	-	<0.001	-

**Table 4:** Dietary diversity scores and meal pattern characteristics

**Dietary variable**	**Low SES (mean ± SD)**	**Middle SES (mean ± SD)**	**High SES (mean ± SD)**	**P-value**
Dietary diversity score (DDS)	4.1 ± 1.0	5.3 ± 1.1	6.3 ± 1.2	<0.001
Green leafy vegetables (servings/week)	1.9 ± 0.6	3.1 ± 0.9	4.0 ± 1.0	<0.001
Pulses & legumes	2.5 ± 0.8	3.4 ± 0.7	4.1 ± 0.8	<0.001
Fruit intake	1.2 ± 0.5	2.4 ± 0.8	3.1 ± 0.9	<0.001
Animal protein intake	0.8 ± 0.3	1.4 ± 0.5	2.2 ± 0.7	<0.001

**Table 5:** Association between dietary diversity and anemia

**Variable**	**Anemia present (n = 162)**	**Anemia absent (n = 174)**	**P-value**
Mean DDS	4.2 ± 1.1	5.9 ± 1.3	<0.001
GLV intake (servings/week)	2.1 ± 0.7	3.5 ± 0.9	<0.001
Fruit intake	1.4 ± 0.6	2.6 ± 0.9	<0.001

**Table 6:** Logistic regression analysis for predictors of anemia

**Predictor variable**	**Adjusted odds ratio (AOR)**	**95% ci**	**P-value**
Low SES	2.71	1.65 - 4.45	<0.001
Poor dietary diversity (DDS <5)	2.39	1.52 - 3.76	<0.001
Female gender	1.34	0.86 - 2.09	0.19
Age (>70 years)	1.22	0.76 - 1.96	0.36

**Table 7:** Comparison of haemoglobin levels across SES categories

**SES Category**	**Mean haemoglobin (g/dl) ± SD**	**P-value**
Low SES	11.2 ± 1.3	<0.001
Middle SES	11.9 ± 1.2	
High SES	12.6 ± 1.1	

## References

[1] https://www.who.int/.

[2] https://www.who.int/news-room/.

[3] Safiri S (2021). J Hematol Oncol..

[4] Greenblum G (2022). J Public Health Emerg..

[5] Wacka E (2024). J Clin Med..

[6] Natekar P (2022). Clin Epidemiol Global Health..

[7] Srivastava S, Kumar S (2021). BMC Public Health..

[8] Retnakumar C (2020). J Family Med Prim Care..

[9] Mandal I, Hossain SR (2024). Int J Community Med Public Health..

[10] Katsumi A (2021). Geriatr Gerontol Int..

[11] SaiHarika B (2025). BMC Geriatr..

[12] Zhang H (2023). BMC Geriatr..

[13] Lopes SO (2022). Int J Environ Res Public Health..

[14] Moradi Y (2025). BMC Public Health..

[15] Bhatnagar RS (2021). Nutrients..

[16] Kumar SB (2022). Nutrients..

[17] Liu L (2024). BMC Geriatr..

